# Neural Representation of Exogenous and Endogenous Temporal Expectations Based on fMRI

**DOI:** 10.1002/cns.70864

**Published:** 2026-04-23

**Authors:** Zhongtian Guan, Zhixi Zhang, Jingyi Zhao, Hongbin Han, Dehua Chui, Jinglong Wu, Qingyuan He, Yixuan Yuan, Wanyi Fu, Xu Zhang, Chunlin Li

**Affiliations:** ^1^ School of Biomedical Engineering Capital Medical University Beijing China; ^2^ Beijing Key Laboratory of Clinical Engineering Solutions for Mental Health Beijing China; ^3^ Aerospace Information Research Institute Chinese Academy of Sciences Beijing China; ^4^ School of Public Health Shandong First Medical University & Shandong Academy of Medical Sciences Jinan China; ^5^ Department of Radiology Peking University Third Hospital Beijing China; ^6^ Neuroscience Research Institute Peking University Third Hospital Beijing China; ^7^ Key Laboratory of Biomimetic Robots and Systems, Ministry of Education Beijing Institute of Technology Beijing China; ^8^ Department of Electronic Engineering The Chinese University of Hong Kong Hong Kong SAR China; ^9^ Institute of Medical Technology Peking University Health Science Center Beijing China; ^10^ Beijing Key Laboratory of Intelligent Neuromodulation and Brain Disorder Treatment Beijing China

**Keywords:** endogenous temporal expectation, exogenous temporal expectation, explicit timing, fMRI, implicit timing

## Abstract

**Aims:**

Temporal expectations are considered as implicit timing, which is different from explicit timing. Furthermore, temporal expectations could be divided into exogenous and endogenous temporal expectations. However, it is still unclear about the neural activation under temporal expectations.

**Methods:**

In the present study, an experimental paradigm was designed for eliciting the related brain activation under exogenous temporal expectations. Three conditions were used for the exogenous temporal expectations task. In order to compare the exogenous temporal expectations related activations to the endogenous', a proper endogenous temporal expectations task was used. Brain activations were obtained by using functional magnetic resonance imaging (fMRI).

**Results:**

Exogenous temporal perception‐related regions, including TPJ, MTG, thalamus, IFG, caudate, cuneus, SOG, calcarine, FEF, and SPL have a good agreement with previous studies. Furthermore, it shows that the precuneus, PCC, Brodmann area 8 (BA8), ACC, and BA10 were also activated, which overlap with regions of the mesial of the so‐called “default mode network”. Negative correlated activations to exogenous temporal expectations task (use an endogenous temporal expectations task as an analysis baseline) were also evaluated.

**Conclusion:**

We found that the sum of exogenous and endogenous temporal expectations related cerebral regions was almost the same when compared to resting‐state networks (RSNs). We propose that the cerebrum could activate in two modes for cognition: one is based on endogenous temporal expectations, and another is based on exogenous temporal expectations.

## Graphical abstract

fMRI revealed dissociable whole‐brain activation patterns for exogenous and endogenous temporal expectation. Behavior showed distinct temporal‐performance signatures: exogenous cueing produced speed‐graded responses, whereas endogenous expectation relied on internally maintained temporal predictions. Whole‐brain responses were categorized into positive and negative task effects to enable integrated anatomical and network‐level characterization.

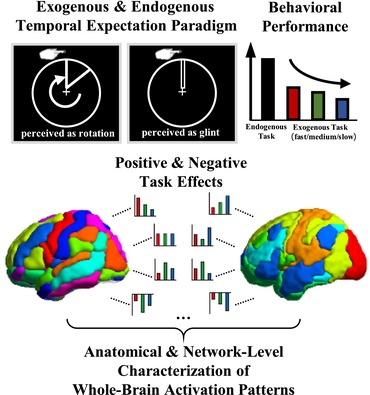

## 1 Introduction

Timing is essential to human behavior, but the neural mechanisms underlying time perception are still unclear [1]. Understanding the timing of events, such as a motor act followed by a sensory consequence, is critical for moving, speaking, determining causality, and decoding the barrage of temporal patterns at our sensory receptors [2]. Researchers [3, 4] indicated that it might be necessary to integrate data from several approaches to reveal the neural mechanisms of interval timing. The evidence supports the idea that there are two timing circuits that can be dissociated: an automatic timing system that works in the millisecond range, which is used in discrete‐event (discontinuous) timing and involves the cerebellum; and a continuous‐event, cognitively controlled timing system that requires attention and involves the basal ganglia and related cortical structures. Because these two timing systems work in parallel, suitable experimental controls might be required to engage (and reveal) each system independently of the other.

Pöppel [5] postulated a dual system for perceiving durations shorter and longer than 2 s. This theory has received plenty of experimental support [6, 7]. A fMRI study [8] by using a perceptual timing (explicit timing) paradigm, which is usually used for investigating time estimation, showed that there are three stages and four neural systems in time estimation in good agreement with Gibbon's scalar expectancy [9]. Furthermore, Morillon et al. [8] concluded that the motor system automatically tracks durations below 2 s, mesial brain regions of the so‐called “default mode network” keep track of longer events. According to Coull and Nobre's classification of time perception [10], this work belongs to explicit time category.

Concretely, Coull and Nobre [10] have divided time perception into two categories, explicit timing and implicit timing. Furthermore, explicit timing was sorted into motor timing and perceptual timing; implicit timing was sorted into emergent timing and temporal expectations, respectively. Finally, temporal expectations were sorted into exogenous temporal expectations and endogenous temporal expectations [10, 11]. The present study focuses on this temporal‐expectation branch by contrasting exogenous and endogenous temporal expectations. According to this classification method, for tasks in which implicit timing is indexed by the temporal regularity of a motor output, timing is said to emerge as a by‐product of the dynamics of motor control (“emergent timing”) [12].

Temporal expectation is increasingly treated as a dynamic form of temporal attention that allocates processing resources to likely moments in time [13]. Temporal prediction can integrate multiple layers of temporal regularities, including unconditional and conditional timing statistics, and these statistics can be encoded in partially separable neural systems that later require integration for behavior [14]. Moreover, the behavioral benefit of exogenous temporal orienting is not fixed but varies with temporal uncertainty, indicating that cue‐driven temporal attention is constrained by the precision of temporal predictions [15]. Consistent with this proactive account, causal evidence further supports a necessary contribution of the cerebellum when temporal expectations are used to tune perceptual sensitivity at the anticipated time [16]. However, direct whole‐brain network‐level evidence from a unified fMRI paradigm that contrasts exogenous and endogenous temporal expectations remains limited.

Therefore, in the present study, we designed a unique experimental paradigm to investigate the timing of exogenous and endogenous temporal expectations and aimed to provide a whole‐brain characterization of their neural correlates. Three kinds of dynamic visual stimulus were used in the exogenous temporal expectations task in which the presentation time of visual stimuli for temporal expectations was set with a length of 400, 800, and 1200 ms, respectively. In order to exclude some brain activations which could be caused by target detection and motor action, further to compare the exogenous temporal expectations related activations to the endogenous, a proper endogenous temporal expectation was used as baseline. Fixed inter‐stimulus interval (ISI) was used to produce endogenous temporal expectations during the control task [10].

## 2 Materials and Methods

### 2.1 Participants

Participants were 23 healthy right‐handed students aged 21–26 years that have normal vision (all participants are males). The protocol was approved by the Ethics Committee of Capital Medical University (2025SY‐045) in accordance with the declaration of Helsinki, and all subjects had given their written informed consent.

### 2.2 Procedures and Task

Visual stimuli were generated on a personal computer and presented to the participants via a custom‐built magnet‐compatible video system during MR scanning. Stimuli were presented from Presentation 22.0 (http://www.neurobs.com/presentation/).

The aim of the exogenous and endogenous temporal expectations tasks is to engage the timing mechanisms automatically (implicitly) rather than deliberately (explicitly), but with an exogenous and endogenous cue, respectively [17]. A blocked design (shown in Figure 2A) was used for the present experiment. 10 trials were carried out continuously in one block for the same condition, but with a randomized order between blocks. For each participant, one session was carried out with a total of 30 trials for each exogenous temporal expectations condition and endogenous temporal expectations task. The total time of the session is 496 s.

**FIGURE 1:**
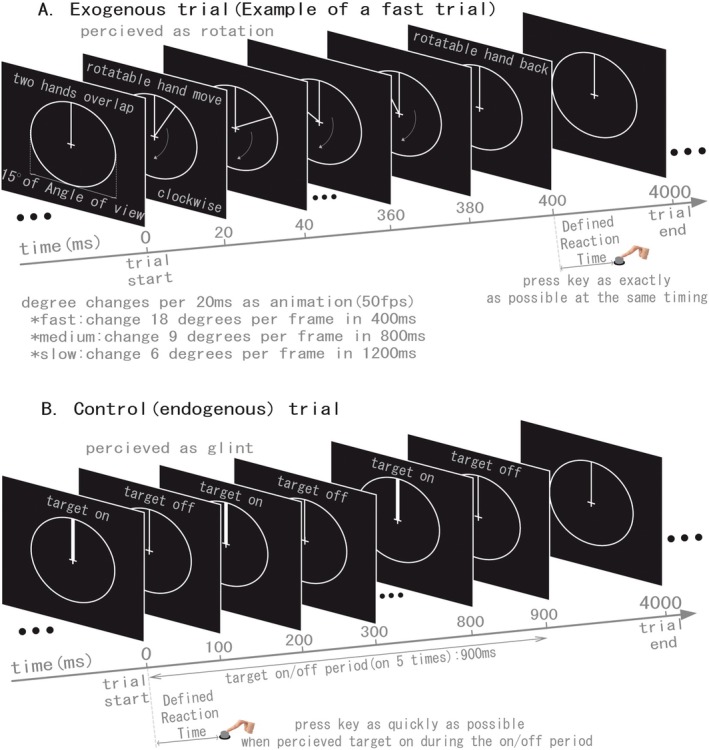
Experiment paradigm. Exogenous trials (A) and Control (endogenous) trials (B) were used during experiments to obtain behavioral data and brain activation. During exogenous trials, participants pressed the reaction key as exactly as possible at the same time when the movable hand backed to the 12 o'clock position. There are three conditions for the exogenous task; the rotation time of the movable hand for one circle was used to define the conditions: 400 ms for fast condition, 800 ms for medium condition, 1200 ms for slow condition. During endogenous trials, the target was presented during the first 900 ms of the trial, and on five times with four times off. Participants perceived the target on/off as a glint and pressed the reaction key as quickly as possible when perceiving the target on.

For exogenous temporal expectations task, a unique paradigm was designed as an implicit temporal perceptual processing [10]. A stimulus was used in order to make the participants think it is a dial (shown in Figure 1A). The diameter of the circle was made with 15 degrees of angle of view from the center of the visual field with a fixation point of a cross. As a background, fixed and moveable hands of the clock overlap at the 12 o'clock position. During a trial of the temporal expectations task, the moveable hand moves clockwise for one circle and back to the 12 o'clock position. There are three conditions for the temporal expectations task. For the fast condition, the moveable hand moves one circle using 400 ms, and 800 ms in the medium condition, 1200 ms in the slow condition, respectively. Animation was used in order to show the hand moves continuously. Components of the animation are figures that show the moveable hand at different angles from the fixed hand (12 o'clock position). In all three conditions, the angle degree changes every 20 milliseconds to ensure that the animation is continuous, because 50 fps (frames per second) in the present study is bigger than a standard 30 fps that is usually used for cartoons. The angle made by the fixed and moveable hands changes eighteen degrees in the fast condition, nine degrees in the medium condition, and six degrees in the slow condition, respectively. During the temporal expectations task, participants were instructed to press the reaction key as exactly as possible when the moveable hand backed to the 12 o'clock position after moving one circle. Thus, the position of the moveable hand was helpful for the temporal expectations, because the same presentation time of every angle degree could give the hint as an exogenous cue [9, 17]. The inter‐stimulus interval (ISI) used in the exogenous temporal expectations task was fixed at 4 s.

For endogenous temporal expectations task, a target detection paradigm was used (Figure 1B). During a trial, hands at the 12 o'clock position turn to wider for 100 ms, and return to quondam width for 100 ms. The turn on and off are carried out for 5 repetitions. Thus, the presentation time from first change of width to 5th lasts 900 ms (5 times on and 4 times off). The reason of the time length of target presentation is to keep the same percentage of time length to the stimulus used in temporal expectations task. The percentage of time length of stimulus presentation was defined as below: during temporal expectations task (400 ms + 800 ms + 1200 ms)/(3 × 4000 ms) = 0.2. It means there are 20% of the time was used for stimulus presentation. Thus, an approximate percentage for stimulus presentation was considered during control task with a premise that target on and off with a duration of 100 ms every time. As a result, 5 times for target presentation in control trial and a 22.5%‐time length for stimulus presentation was calculated. Participants were instructed to press the reaction key as quickly as possible when they saw the target on. It is important that the inter‐stimulus interval (ISI) used in endogenous temporal expectations task was fixed at 4 s to induce the temporal expectations covertly, thus the trial in the same block may play a role of endogenous cue for the next trial [10, 17].

### 2.3 Functional MR Scanning

Images were acquired using a 3‐T Siemens scanner vision whole‐body MRI system to measure the brain activation with a head coil. The imaging area consisted of 32 functional gradient‐echo planar imaging (EPI) axial slices (voxel size 3 × 3 × 4 mm, TR = 4000 ms, TE = 50 ms, FA = 90°, 128 × 128 matrix) that were used to obtain T2*‐weighted fMRI images in the axial plane. For each participant, we obtained 124 functional volumes.

### 2.4 Behavioral Data Analysis

RTs (Reaction times) were used as behavioral data. For each exogenous temporal expectations conditions condition and endogenous temporal expectations task, there are ~690 RTs for statistical analysis. The RT data during the fMRI experiment were analyzed using one‐way repeated measure ANOVA with 4 levels with equal variance assumption (SPSS 16.0 for Windows). LSD (least significant difference) multiple comparison at *p* < 0.05 was used for the post hoc tests for the pair‐wise comparisons.

### 2.5 fMRI Data Analysis

For the functional image analysis, we first used MRIcron (https://www.nitrc.org/projects/mricro) to convert the DICOM files to NIFTI files. The first four functional images were discarded because of the changeful contrast of the images.

Data preprocessing and statistical analyses were performed with the Statistical Parametric Mapping computer package (SPM12, http://www.fil.ion.ucl.ac.uk/spm/) implemented in MATLAB 2018a. All volumes were realigned spatially to the first volume of the first time series. The movement parameters generated during spatial realignment indicated that all 23 participants moved less than 2 mm. Realigned images were spatially normalized using the standard EPI template in the Montreal Neurological Institute (MNI) reference brain coordinate space [18] and re‐sampled into 2 × 2 × 2 mm voxels [19]. Normalized images were smoothed with an isotropic 8 mm FWHM (full‐width half maximum) Gaussian kernel.

Statistical analysis was performed in two stages of a mixed‐effects model. In the first‐level analysis, the BOLD response was modeled as the neural activity convolved with a canonical hemodynamic response function (HRF) [20] to yield regressors in a general linear model (GLM) for each condition (Fast vs. Endogenous task, Medium vs. Endogenous task, and Slow vs. Endogenous task). The time series in each voxel were high‐pass filtered to remove low‐frequency noise and scaled within session to a grand mean of 128. Non‐sphericity of the error covariance was accommodated by an AR (1) (first‐order autoregressive) model, in which the temporal autocorrelation was estimated by pooling over suprathreshold voxels [21].

The “con” or contrast images of the first‐level analysis from all 23 subjects were then used for the second‐level group statistics. To identify the whole brain activation for time expectations, one‐way repeated measure analysis of variance (ANOVA) was used to examine average positive and negative activations of the 3 conditions when compared to the endogenous task. Only effects surviving an uncorrected threshold of *p* < 0.005 and including 25 or more contiguous voxels were interpreted for the average positive activation, and FWE (family wise error) corrected threshold of *p* < 0.05 and including 5 or more contiguous voxels were interpreted for the average negative activation. Three masks (made from contrast of condition fast, medium, slow, respectively) were used for obtaining the negative (exclusive) activations. For further confirmation of activations, low‐level of *p* values (0.01 uncorrected for positive, 0.001 uncorrected for negative) were used to elicit some other related activations.

In order to further evaluate the significant difference of regional signal change between the three conditions, the ROI (region of interest) analysis was carried out. Center of sphere regions were defined from the SPM coordinates and an 8 mm radius was used in order to keep consistency with the FWHM used in smooth. Then the defined regions were used to extract the averaged data out of the first level individual subject statistical analysis. For each ROI, there were a total of 69 measurements from 3 conditions of all 23 participants. Data were then used in repeated measures analysis of variance (ANOVA; SPSS 16.0 for Windows) with equal variances assumption. LSD (least significant difference) at *p* < 0.05 was used for the post hoc multiple comparison to test the pair‐wise comparisons.

## 3 Results

### 3.1 Behavioral Results

RT (Reaction time) during the exogenous temporal expectations conditions was defined by the time difference between the timing of moveable hand backs to the 12 o'clock position and the timing of key pressing. We excluded the RT when the participant pressed the key not only before the start of the trial but also later more than 1 s after the timing of moveable hand backs to the 12 o'clock position. All RTs from participants obtained during the exogenous temporal expectations task had a high adoption ratio with an average percentage of 98%, but one participant had 80%. RT during the endogenous temporal expectations was defined by the time difference between the timing of the first time of target on and the timing of key pressing. We excluded the RT when it was not only smaller than 100 ms but also bigger than 1 s. All RTs from participants obtained during the endogenous temporal expectations task also had a high adoption ratio with an average percentage of 90%.

**FIGURE 2:**
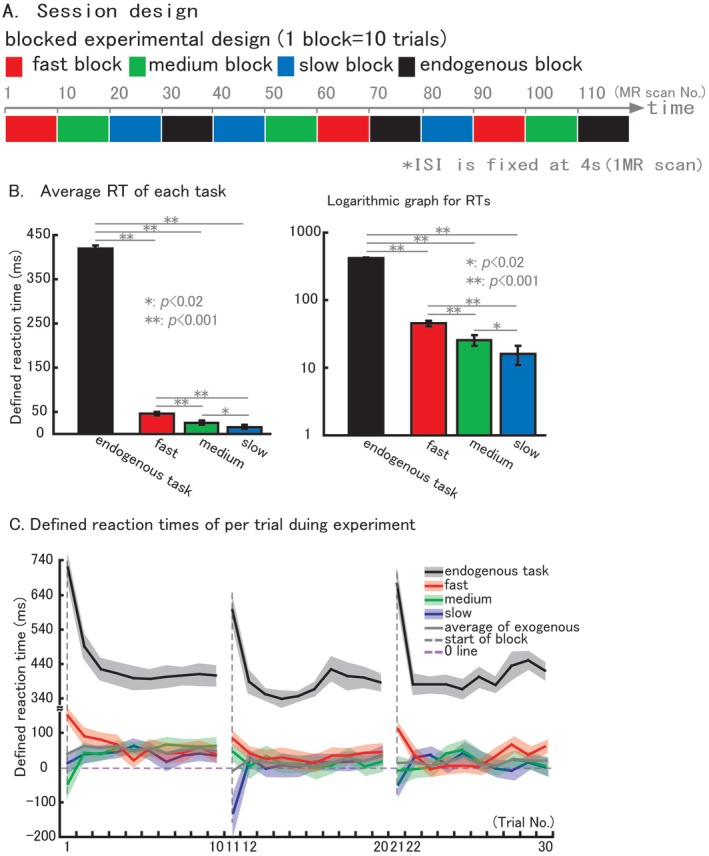
Behavioral results. Blocked experimental design (A) was used. During experiment session, same condition was carried out 10 trials continuously. Endogenous block was fixed with equidistance, and three types of exogenous blocks were pseudorandom. (B) Average defined reaction times (RTs) for each exogenous condition and endogenous task. Logarithmic graph was used because of the large gap of defined RTs between endogenous and exogenous trials. (C) Defined reaction times of per trial for each condition. Black line shows trend of RTs between trials in endogenous task. Red line shows trend of RTs between trials in fast condition of exogenous task. Green line shows trend of RTs between trials in medium condition of exogenous task. Blue line shows trend of RTs between trials in slow condition of exogenous task. Gray line shows trend of RTs between trials of average of exogenous trials. Vertical dashed line (gray) means the start of each block. Horizontal dashed line (purple) means the defined RT datum line. Pairwise comparisons results are shown in the histogram (*p < 0.02, **p < 0.001). The error bar is shown in standard error (SE).

As shown in Figure 2B, the difference of averaged RT between the exogenous temporal expectations conditions and endogenous temporal expectations task with a large gap. For visualization in the logarithmic plot, RT values were converted to their absolute values. 420 ms with an SE (standard error) of 6.7 during endogenous temporal expectations, 45 ms (SE = 4.4) for fast, 26 ms (SE = 4.7) for medium, 16 ms (SE = 5.1) for slow, respectively. Furthermore, pair‐wise comparisons of statistical analysis showed that, not only the exogenous temporal expectations conditions when compared to the endogenous temporal expectations task have a significant difference (all of the three pairs showed *p* < 0.001), but also the pairs among the three conditions showed significant difference with each other (fast vs. medium: *p* < 0.001; fast vs. slow: *p* < 0.001; medium vs. slow: *p* < 0.02). Figure 2C shows the defined reaction times of per trial during experiment. Black line shows the averaged RT for each trial during endogenous temporal expectations task, a curve with a trend of turning to lower in right, was produced by the large difference between the first trial and the rest 9 trials in the same block. The same trend was also showed from fast condition (red line), but a reversed trend was obtained from medium condition (green line) and slow condition (blue line). Gray line shows the averaged trend of the three conditions trial‐by‐trial. Red line (fast condition) shows a similar trend when compare to black line (endogenous task). Exogenous temporal expectation showed speed‐graded, near‐zero responses defined relative to the clock‐hand return, whereas endogenous temporal expectation showed substantially longer detection RTs and a block‐wise trial effect driven mainly by the first versus subsequent trials.

### 3.2 Functional Imaging Results

#### 3.2.1 High‐Level Positive Effect to Exogenous Temporal Expectations

**FIGURE 3:**
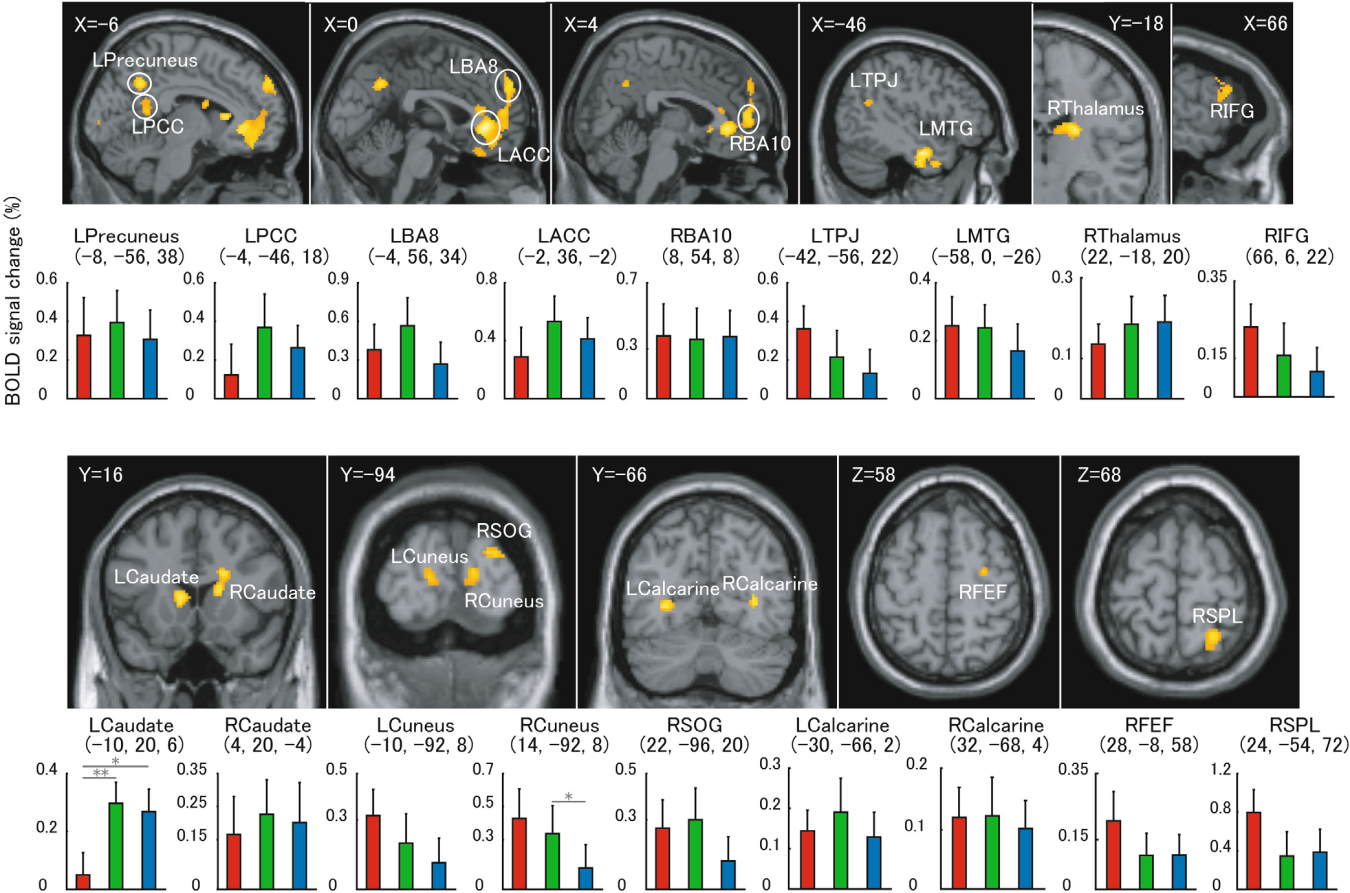
High‐level positive effect during exogenous trials is shown as section results and Blood Oxygen level‐dependent signal change results. Section results were elicited under a threshold of uncorrected *p* < 0.005 with a cluster level over 25 voxels. Centre of each region of interest was defined by peak voxel of each anatomical region, and 8 mm radius was used for each ROI. R: Right hemisphere; L: Left hemisphere; PCC: Posterior cingulate cortex; BA8: Brodmann area 8; ACC: Anterior cingulated cortex; BA10: Brodmann area 10; TPJ: Temporoparietal junction; MTG: Middle temporal gyrus; IFG: Inferior frontal gyrus; SOG: Superior occipital gyrus; FEF: Frontal eye field; SPL: Superior parietal lobe. Red stick, green stick and blue stick in histogram mean fast condition, medium condition, and slow condition, respectively. Pairwise comparisons results are shown in the histogram (*p < 0.05; **p < 0.01). The error bar is shown in standard error (SE).

Figure 3 shows the mainly section results of averaged positive activation during temporal expectations conditions and ROI‐based BOLD (blood oxygen‐level dependent) signal change for each condition. Table 1 shows the summarized whole brain activations. As shown in Figure 3, we confirmed several cortical and sub‐cortical regions under a threshold of *p* < 0.005, uncorrected. Precuneus and PCC (posterior cingulate cortex) (section result with X = −6), BA8 (Brodmann area 8) and ACC (anterior cingulated cortex) (section result with X = 0), BA10/11 (Brodmann area 10/11) (section result with X = 4), have an overlapping with regions of the so‐called “default mode network” [22, 23]. Temporal perception related regions, TPJ (temporoparietal junction) [24] and MTG (middle temporal gyrus, section result with X = −46) and thalamus [8] (section result with Y = −18), IFG [25] (inferior frontal gyrus) (section result with X = 66), caudate [17, 26] (section result with Y = 20), Cuneus and SOG (superior occipital gyrus) [25] (section result with Y = −94), calcarine [27] (section result with Y = −66), FEF (frontal eye field) [25] (section result with Z = 58), SPL (superior parietal lobe) [28] (section result with Z = 68). ROI‐based BOLD signal change for each condition does show wide spread significant difference across these regions, but within left caudate and right cuneus (see Figure 3). Cerebellum seems to be a subsidiary role in the present study, only shows activation under a smaller cluster size and lower threshold level (*p* < 0.05, uncorrected), and three clusters were observed: (1) cluster size = 127, peak voxels: −14, −58, −28, *p* < 0.005; (2) cluster size = 36, peak voxels: 20, −58, −28, *p* < 0.005; (3) cluster size = 46 voxels, peak voxels: −16, −44, −38, *p* < 0.006.

**TABLE 1:** Summarized brain activations of high‐level positive effect for exogenous time expectations.

Cluster size (voxels)	Anatomy region	*Z* score	*x*	*y*	*z*
566	L MTG	4.69	−58	0	−26
1431	L ACC	4.55	−2	36	−2
30	L Calcarine	4.13	−30	−66	2
	R BA10/11	3.69	8	54	8
	L BA8	3.58	−4	56	34
189	R Thalamus	4.14	22	−18	20
122	L Caudate	3.93	−10	20	6
358	R Caudate	3.85	4	20	−4
192	L Precuneus	3.83	−8	−56	38
125	R SPL	3.72	24	−54	72
34	R SOG	3.56	22	−96	20
10	R Calcarine	3.41	32	−68	4
69	R Cuneus	3.37	14	−92	8
44	R IFG	3.24	66	6	22
28	R FEF	3.23	28	−8	5
56	L Cuneus	3.22	−10	−92	8
27	R OFC	3.18	2	34	−26
54	L TPJ	3.16	−42	−56	22
381	L PCC	2.69	−4	−46	18

*Note:* The approximate anatomical regions are from the Talairach atlas, and the *x*, *y*, and *z* coordinates are from SPM12.

Abbreviations: L, Left hemisphere; R, Right hemisphere.

#### 3.2.2 High‐Level Negative Effect to Exogenous Temporal Expectations

**FIGURE 4:**
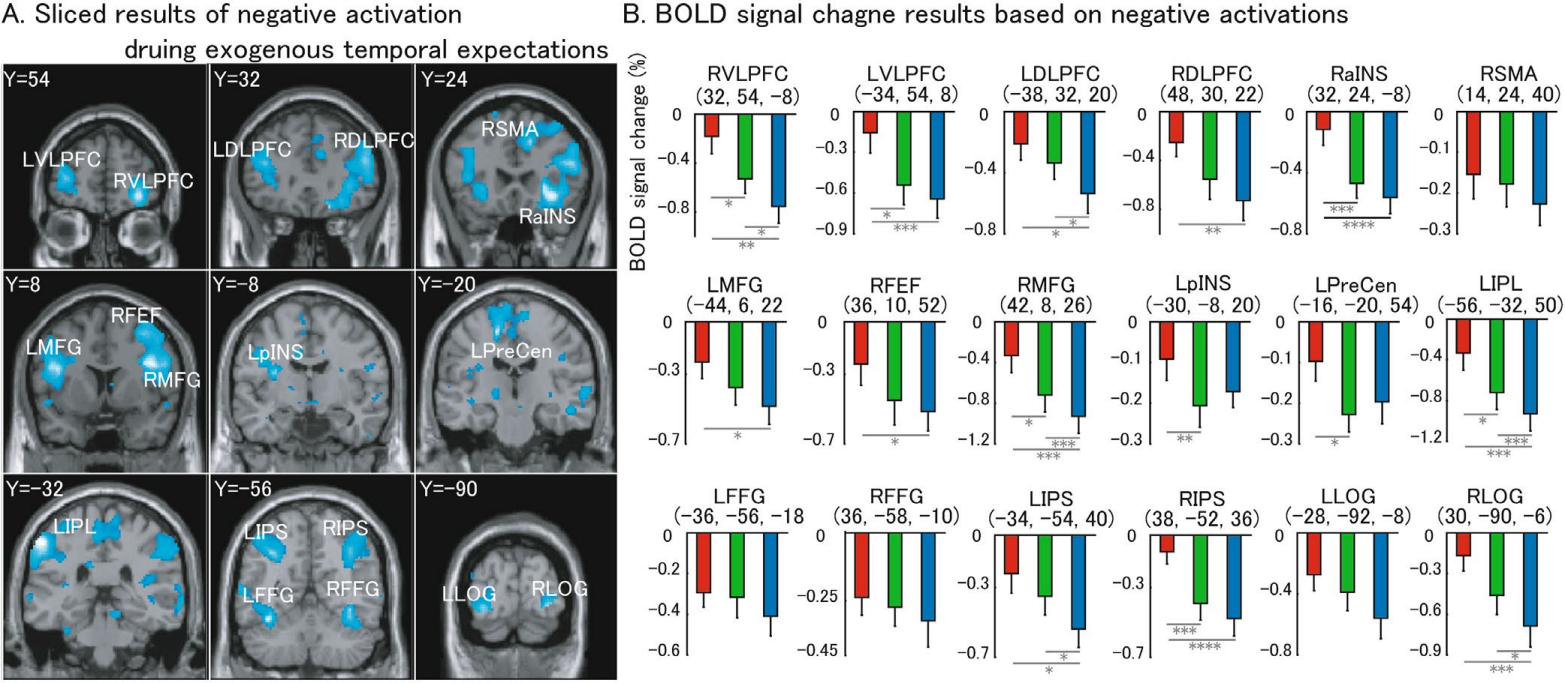
High‐level negative effect during exogenous trials is shown as section results and Blood Oxygen level‐dependent signal change results. Section results were elicited under a threshold of family wise error (FWE) *p* < 0.05 with a cluster level over 5 voxels. Centre of each region of interest was defined by peak voxel of each anatomical region, and 8 mm radius was used for each ROI. R: Right hemisphere; L: Left hemisphere; VLPFC: Ventrolateral prefrontal cortex; DLPFC: Dorsolateral prefrontal cortex; SMA: Supplementary motor area; aINS: Anterior insula; MFG: Middle frontal gyrus; FEF: Frontal eye field; pINS: Posterior insula; PreCen: Precentral sulcus; IPL: Inferior parietal lobe; IPS: Inferior parietal sulcus; FFG: Fusiform gyrus; LOG: Lateral occipital gyrus. Red stick, green stick and blue stick in histogram mean fast condition, medium condition, and slow condition, respectively. Pairwise comparisons results are shown in the histogram (*p < 0.05; **p < 0.01; ***p < 0.005; ****p < 0.001). The error bar is shown in standard error (SE).

Figure 4 shows the mainly section results of averaged negative activation during temporal expectations conditions and ROI‐based BOLD (blood oxygen‐level dependent) signal change for each condition. Because we used a target detection paradigm as the control task, these negative activations are considered to have a positive correlation with the target detection process. Table 2 shows the summarized whole brain activations. As shown in Figure 4, we confirmed several cortical regions under a threshold of *p* < 0.05, with FWE correction. VLPFC (ventrolateral prefrontal cortex) (section result with Y = 54), DLPFC (dorsolateral prefrontal cortex) (section result with Y = 32), SMA (premedial motor cortex) and aINS (anterior insula) (section result with Y = 24), MFG (middle frontal gyrus) and FEF (frontal eye field) (section result with Y = 8), pINS (posterior insula) (section result with Y = −8), PreCen (precentral cortex) (section result with Y = −20), IPL (section result with Y = −32), IPS (inferior parietal sulcus) and FFG (fusiform gyrus) (section result with Y = −56), LOG (lateral occipital gyrus) (section result with Y = −90). ROI‐based BOLD signal change for each condition was shown as negative signal for temporal expectations task. Within the majority of those regions, signal change shows a significant difference across the three conditions.

**TABLE 2:** Summarized brain activations of high‐level negative effect for exogenous time expectations.

Cluster size (voxels)	Anatomy region	*Z* score	*x*	*y*	*z*
251	L IPL	6.20	−56	−32	50
415	R MFG	5.97	42	8	26
	R DLPFC	5.45	48	30	22
49	L FFG	5.93	−36	−56	−18
62	R IPS	5.89	38	−52	36
193	R aINS	5.87	32	24	−8
45	L PreCen	5.67	−16	−20	54
128	L MFG	5.63	−44	6	22
88	R VLPFC	5.60	32	54	−8
30	L LOG	5.51	−28	−92	−8
24	R FFG	5.33	36	−58	−10
10	R LOG	5.32	30	−90	−6
10	R SMA	5.30	14	24	40
5	R OFC	5.29	18	32	−18
45	L IPS	5.24	−34	−54	40
9	L pINS	5.12	−30	−8	20
17	R FEF	4.99	36	10	52
5	L VLPFC	4.95	−34	54	8
10	L DLPFC	4.93	−38	32	20

*Note:* The approximate anatomical regions are from the Talairach atlas, and the *x*, *y*, and *z* coordinates are from SPM12.

Abbreviations: L, Left hemisphere; R, Right hemisphere.

#### 3.2.3 Low‐Level Positive and Negative Effect to Exogenous Temporal Expectations

**FIGURE 5:**
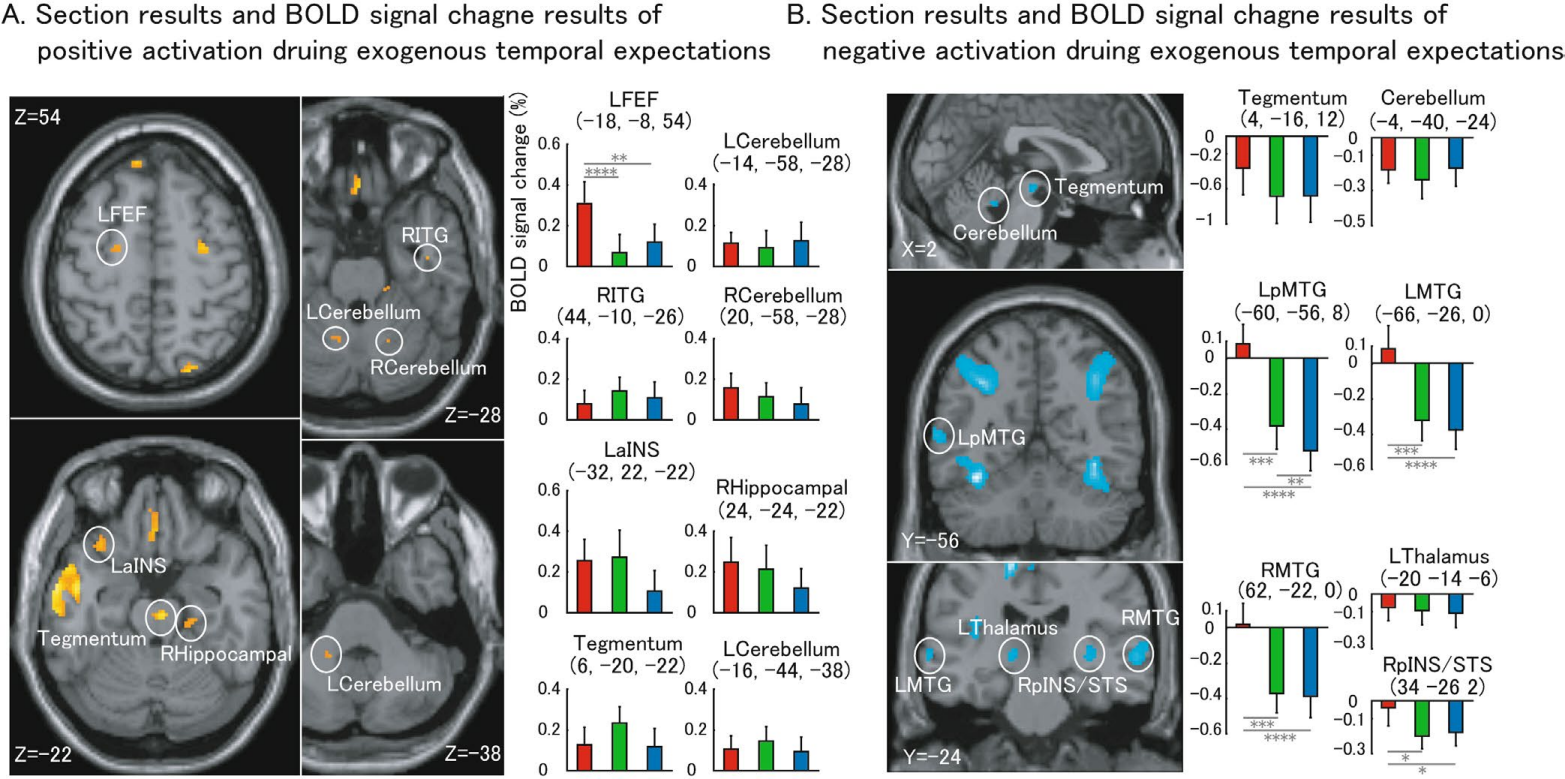
Low‐level positive and negative effect during exogenous trials. A. Section results and Blood Oxygen level‐dependent signal change results of low‐level positive. B. Section results and BOLD signal change results of low‐level negative. Section results were elicited under a threshold of uncorrected *p* < 0.01 for positive effect and uncorrected *p* < 0.001 for negative effect, respectively. Centre of each region of interest was defined by peak voxel of each anatomical region, and 8 mm radius was used for each ROI. R: Right hemisphere; L: Left hemisphere; FEF: Frontal eye field; ITG: Inferior temporal gyrus; aINS: Anterior insula. Red stick, green stick and blue stick in histogram mean fast condition, medium condition, and slow condition, respectively. Pairwise comparisons results are shown in the histogram (*p < 0.05; **p < 0.01; ***p < 0.005; ****p < 0.001). The error bar is shown in standard error (SE).

When threshold was down to a low‐level, some regions appeared distinctly. Figure 5 shows the low‐level effect both from positive (A) and negative (B). Left FEF, Left aINS [26], Right ITG [8], Right Hippocampal [29], Cerebellum [30] and Tegmentum were observed from positive effect to exogenous temporal expectations. Left pMTG, Left MTG, Right MTG, Right pINS/STG, Left Thalamus, Cerebellum [8] and Tegmentum [31] were observed from negative effect to exogenous temporal expectations. Summarized related regions are shown in Table 3.

**TABLE 3:** Summarized brain activations of low‐level positive and negative effect during exogenous trials.

Cluster size (voxels)	Anatomy region	*Z* score	*x*	*y*	*z*
Positive					
25	Tegmentum	3.72	6	−20	−22
30	L aINS	2.85	−32	22	−22
19	R Hippocampal	2.69	24	−24	−22
17	L FEF	2.60	−18	−8	54
2	R Cerebellum	2.59	20	−58	−28
11	L Cerebellum	2.58	−14	−58	−28
3	R ITG	2.58	44	−10	−26
3	L Cerebellum	2.53	−16	−44	−38
Negative					
241	R MTG	4.67	62	−22	0
57	R pINS/STS	4.49	34	−26	2
65	L pMTG	4.30	−60	−56	8
34	L MTG	4.26	−66	−26	0
34	Tegmentum	4.19	4	−16	−12
50	L Thalamus	4.04	−20	−14	−6
31	Cerebellum	3.74	−4	−40	−24

*Note:* The approximate anatomical regions are from the Talairach atlas, and the x, y, and z coordinates are from SPM12.

Abbreviations: L, Left hemisphere; R, Right hemisphere.

## 4 Discussion

Temporal expectation can be framed as temporal orienting of attention, whereby processing is prioritized at behaviorally relevant moments [11, 25, 32]. In our exogenous paradigm, the continuously evolving position of the moving clock hand provides moment‐to‐moment time‐to‐event information and thus serves as a perceptually available external temporal cue, biasing temporal orienting in a relatively stimulus‐driven manner [25, 32]. In our endogenous paradigm, there is no comparable dynamic temporal cue during the ISI; instead, timing predictability is carried by the fixed ISI across trials, such that temporal expectation is induced without an overt within‐interval cue and supported by internally monitored elapsed time to anticipate upcoming trial onset/event timing and learned temporal regularity within a block [11]. More generally, temporal predictions can arise from temporal regularities/statistics across trials, and such regularity‐based expectations have been reported to differ from explicit cue‐based temporal expectations in other paradigms [33]. Voluntary temporal attention can also be dissociated from timing predictability per se [34]. We use this operational distinction to interpret dissociable neural signatures across the two paradigms. Within this framework, our exogenous task is intended to reflect behavioral performance in temporal orienting supported by continuously available external time‐to‐event information from the moving clock hand, whereas our endogenous task is intended to reflect regularity‐based temporal expectation supported by internally monitored elapsed time across a fixed ISI in the absence of a dynamic within‐interval cue. We further discuss the neural functional representations of these two modes:

### 4.1 Exogenous Temporal Expectations Related Activations

Brain activations related to implicit timing were investigated usually by using a pre‐cue [25, 35]. There is also another kind of study on investigation of implicit timing which uses the velocity of a dynamic visual stimulus [30]. According to the view of Coull et al. [17], both of the two kinds could make temporal predictions. The temporal expectations experimental paradigm that is used in the current study are more similar to the latter.

The DMN underpins task‐independent self‐referenced cognition, memory [36], and is usually deactivated during sensory stimulation [37]. The DMN has never been related to time perception, probably because it is activated during baseline conditions that are usually subtracted out in functional neuroimaging studies [22] or during tasks that do not require focal attention. Lesions to this large network are rare and induce global apathy and incapacity to organize actions [38], rather than specific cognitive impairments. Estimation of long‐time intervals is necessary to plan high‐level behavior, and its impairment is a plausible silent clinical correlate of DMN lesions. Ventral temporal regions close to the comparing system were activated, suggesting that the temporal cortex is also involved in temporal distortions [39]. Yet, as exogenous temporal expectations were also associated with activations in mesial wall regions of the DMN, the latter might also participate in the detection of temporal duration.

Electrophysiological recordings in monkeys [40] show that neural firing varies dynamically as a function of the conditional probability that a target will occur at a particular time, given that it has not already occurred (the “hazard function”). This firing pattern has been observed in visual [27] primary motor [41] and parietal cortices [42] for color, motor and spatial tasks, respectively, indicative of context‐dependent and functionally localized representations of temporal expectations. In support of this, magnetoencephalographic [43] recordings during a choice reaction‐time (RT) task in humans showed increased activity in parietal cortex and cerebellum as a function of the hazard function [44], while increased phase synchronization between parietal cortex, cerebellum and subcortical structures was evident during motor synchronization to temporally predictable (isochronous) rather than random ISIs [28]. fMRI studies using both simple [45] and choice RT [29, 46] have identified increased activity in left premotor and inferior parietal cortices for temporally predictable rather than random ISIs. In addition, synchronizing motor responses to metrically salient (i.e., temporally predictable [47] auditory rhythms increased activity in dorsal premotor cortex [48]). Premotor cortex, inferior parietal cortex and cerebellum form selective action circuits [49], and it appears that temporal expectation simply modulates their motor induced activity.

Our finding substantiates the development of the striatal‐beat frequency (SBF) model for interval timing, which highlights the input–output relationship between the dorsal striatum and other brain regions. In essence, the SBF model states that striatal neurons receive numerous cortical inputs, and then integrate and fire action potentials if the input signals pass a predetermined threshold or reach a specific ensemble firing pattern [50]. Once the neural computation is complete, the dorsal striatum sends a signal to other BG regions, and the BG, in turn, send signals back to the frontal cortex (e.g., motor cortex) through the thalamus.

Specifically, posterior regions of the lateral cerebellum were selectively activated by temporal, when compared with spatial, predictions when subjects used either the velocity of a dynamic visual stimulus [30] or symbolic temporal pre‐cues [35] to make temporal predictions. The cerebellum is thought to use forward modeling to predict the sensory consequences of motor behavior [51]. These fMRI results show that the posterior cerebellum is also involved in the forward modeling of purely perceptual stimuli, but only when the timing of the perceptual stimulus must be predicted.

Task order predictability also activated the right hippocampus and the posterior cingulate gyrus. As mentioned in the Introduction, it has been proposed that retrieving which task has to be performed constitutes the essential part of what subjects can do to prepare before stimulus presentation [52]. The hippocampal formation targets the medial PFC in monkeys [53, 54] and is linked to the medial extension of the mid‐DLPFC via the cingulum bundle [55]. There is evidence that the hippocampus is involved in remembering the sequential order of events [56, 57] and the posterior cingulate cortex has been found activated during various memory retrieval tasks [43, 58]. The asymmetric, right‐sided activation of the hippocampus could be attributed to the nonverbal aspects of the retrieved memory [59].

**FIGURE 6:**
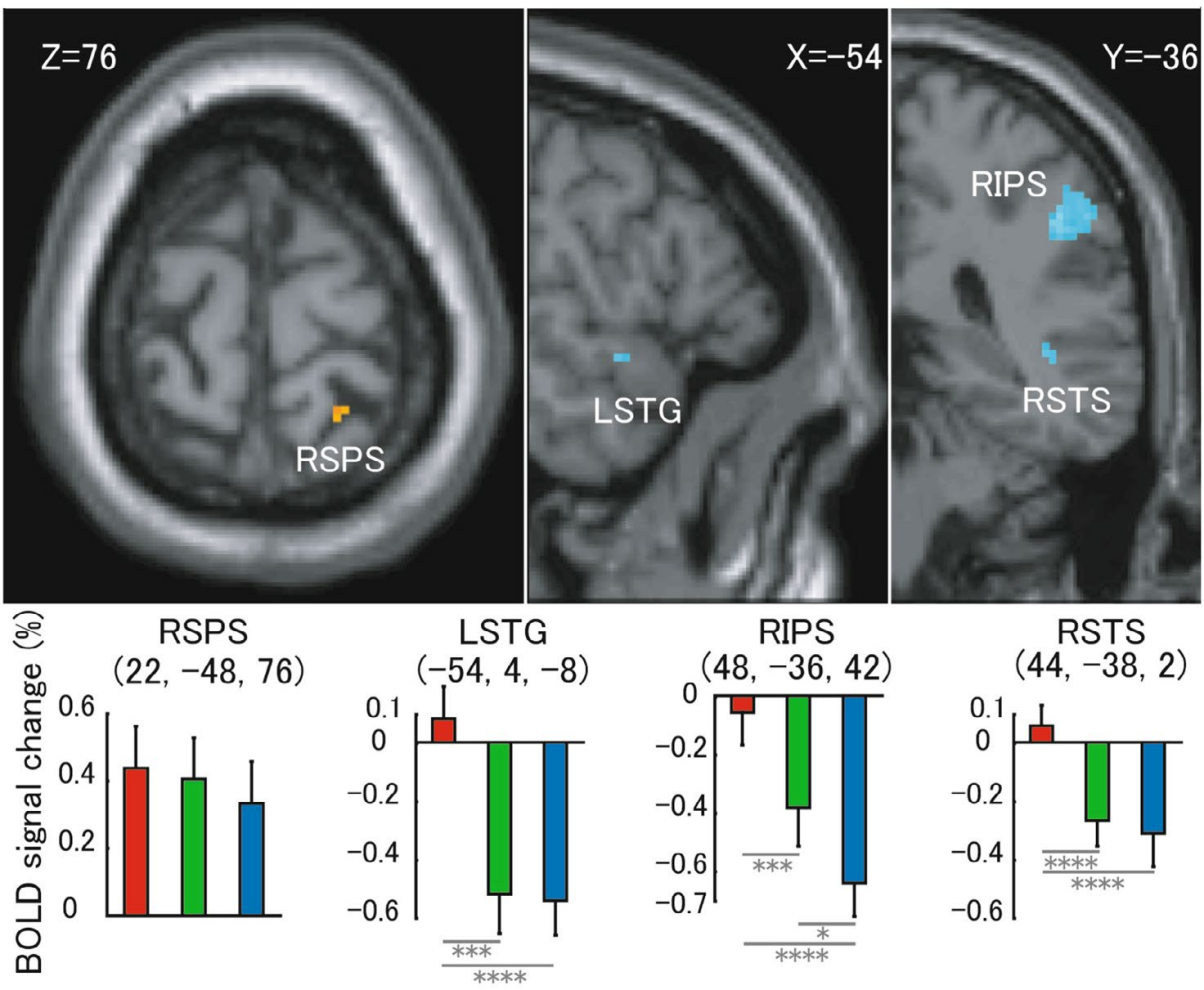
Positive and negative effect during exogenous medium and slow trials. Section results were elicited under a threshold of uncorrected *p* < 0.005 for positive effect and family wise error (FWE) *p* < 0.05 for negative effect, respectively. Centre of each region of interest was defined by peak voxel of each anatomical region, and 8 mm radius was used for each ROI of Blood Oxygen level‐dependent signal change results. R: Right hemisphere; L: Left hemisphere; SPS: Superior parietal sulcus; STG: Superior temporal gyrus; IPS: Inferior parietal sulcus; STS: Superior temporal sulcus. Red stick, green stick and blue stick in histogram mean fast condition, medium condition, and slow condition, respectively. Pairwise comparisons results are shown in the histogram (*p < 0.05; ***p < 0.005; ****p < 0.001). The error bar is shown in standard error (SE).

Regarding the similar trend of behavioral results between fast condition and endogenous task, we considered that perhaps brain activation would change when using the medium and slow conditions for observing the exogenous temporal related neural substrates. Figure 6 shows both positive (*p* < 0.005, uncorrected) and negative (*p* < 0.05, FWE) effects to exogenous conditions (medium and slow). Right SPS showed a positive effect. Left TPJ, Right IFG, bilateral cuneus, Right FEF, and Right SPL disappeared when compared to the results shown in Figure 5 using a more stringent threshold. These regions showed a common trend of BOLD signal, where the fast condition induced the highest level and the slow condition. It implies that these regions may not relate to exogenous temporal expectations but with a function of retinotopy within visual regions [60] and spatial attention within SPL, FEF, and IFG [61, 62]. Right IPS, Right STS, and Left STG showed a negative effect. Increased negative effect regions imply that those regions may also be involved in endogenous temporal expectations.

### 4.2 Endogenous Temporal Expectations Related Activations

Although Cui et al. [63] have well described by the simplest model in which brain activity evolves linearly with time, Coull [64] indicated that the inclusion of an additional factor, the hazard function, explained the data much more completely. The differential explanatory power of the hazard function versus linear models indicates that these cortical effects represent something more akin to the updating of temporal expectations as a function of evolving conditional probabilities, rather than simple estimation of time‐in‐passing. Current negative results (shown in Figures 4 and 5B) showed a linear trend of BOLD signal between exogenous conditions which regions involved in endogenous task. Furthermore, the more endogenous expectations were needed, the stronger activation was observed (difference of BOLD signal when compared to exogenous conditions). We considered that hazard function indeed worked during endogenous task.

Other electrophysiological studies of implicit timing (the hazard function) in monkeys have suggested a more context‐dependent representation of implicit timing in a variety of cortical areas. For example, neural activity was shown to vary dynamically as a function of elapsing time and the likelihood of target onset in early visual cortex in a visual discrimination task [27], in primary motor cortex in a pointing task [41], and in parietal cortex in a spatial saccade task [42]. Whether striatal or SMA neurons also fired simultaneously with neurons in each of these areas, across different task contexts, is of course unknown (Evidence [65] suggests that frontal activity also follows the hazard function). It is important to note, however, that the timing of cortical activity, in the motor cortex at least, seems to reflect the scalar property of interval timing [66, 67], thereby showing timescale invariance, that is, ‘time is like a rubber band' [68].

When fixed temporal expectations have been used to optimize response speed (e.g., by temporally informative cues or temporally predictable sequences), activation of left‐lateralized parietal and ventral premotor circuits is observed [35]. Rao et al. [26] previously showed that the posterior temporal cortex encodes duration. Cued reaction‐time tasks [25], showing that right prefrontal cortex was necessarily implicated in the monitoring or updating of changing conditional probabilities over time.

### 4.3 Resting‐State Networks and Temporal Expectations

With regard to the view of ‘time is of the essence' [30], we considered that exogenous plus endogenous temporal expectations might be involved in almost all of the cortex regions, although these regions may have discrete functions. According to the trend of ROI‐based signal change of the three exogenous temporal expectations conditions, we divided the positive and negative cortical and sub‐cortical regions into ten patterns (Table 4).

**TABLE 4:** Classifications of Blood Oxygen level‐dependent signal change by the trend of three exogenous conditions.

A. Positive effect to exogenous		B. Negative effect to exogenous		C. Neocortex classification by Tensor‐PICA estimation		D. Functional connectivity based on resting‐state‐network parcellation	
a. 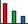	L TPJ L MTG L Cuneus R Hippocampal R Cerebellum R Cuneus R IFG	i. 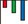	L VLPFC L DLPFC L MFG L Thalamus L MTG L pMTG L IPL L FFG L IPS L LOG R VLPFC R DLPFC R aINS R SMA R MFG R FEF R MTG R FFG R IPS R LOG	A	R SOG^h^ L FFG^i^ L LOG^i^ R FFG^i^ R LOG^i^	A′	R SOG^h^ L FFG^i^ L LOG^i^ R FFG^i^ R LOG^i^ R Cuneus^a^ L Cuneus^a^
b. 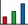	R Thalamus			B	L Precuneus^h^ L PCC^g^ R IPS^i^ L IPS^i^		
c. 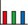	R BA10			C	L FEF^e^ L IPL^i^ L VLPFC^i^ L DLPFC^i^	B′	L pINS^j^ R pINS/STS^j^ L PreCen^j^ R SMA^i^
d. 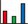	L Cerebellum			D	R SPL^f^ R FEF R DLPFC^i^ R VLPFC^i^ R FEF	C′	L BA8^h^ R FEF L FEF^e^ R SPL^f^ L IPL^i^ R MFG^i^ L MFG^i^ R FEF L pMTG^i^
e. 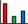	L FEF			E	L Calcarine^h^ R Calcarine^h^ R Cuneus^a^ L Cuneus^a^		
f. 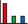	R FEF R SPL	j. 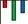	L PreCen L pINS R pINS/STS Tegmentum Cerebellum	F	R IFG^a^ R MFG^i^ L MFG^i^ L pINS^j^	D′	R IFG^a^ L TPJ^a^ R aINS^i^ L aINS^h^
g. 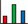	L Caudate R Caudate R ITG L PCC L ACC			G	R MTG^i^ L MTG L TPJ^a^	E′	R ITG^g^ R Hippocampal^a^
h. 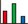	L BA8 L aINS L Precuneus L Calcarine R SOG R Calcarine Tegmentum L Cerebellum			H	L pMTG^i^ L PreCen^j^	F′	R DLPFC^i^ L VLPFC^i^ R VLPFC^i^ L DLPFC^i^ R IPS^i^ L IPS^i^
				I	L aINS^h^ R pINS/STS^j^ R aINS^i^ R SMA^i^	G′	L MTG L ACC^g^ R BA10^c^ L Precuneus^h^ L PCC^g^ R MTG^i^ L MTG
				J	L ACC^g^ R BA10^c^ L BA8^h^		

*Note:* A. Positive effects were divided into 8 types. B. Negative effects were divided into 2 types. C. Neocortex classification based on a previous Tensor‐PICA estimation. Damoiseaux et al. [69] sorted the neocortex into 10 components by using Tensor‐PICA estimation (A–J). Note that temporal expectations employed whole neocortex. D. Delineation of functional networks (A′–G′, Yeo 7 network atlas) in the cerebral cortex using intrinsic functional connectivity MRI based on the results of Yeo et al. [70]. We labeled the type of activation of brain regions simultaneously in both neocortex classifications, in addition to left MTG and right FEF.

At the whole‐brain level, the two modes of temporal expectation exhibited partially dissociable large‐scale network profiles. Endogenous temporal expectation preferentially engaged midline association hubs commonly linked to the default mode network, together with temporo‐parietal and temporal association areas and subcortical timing‐related nodes. In contrast, the complementary pattern distinguishing the exogenous mode was expressed mainly as relative decreases in lateral prefrontal, insular, premotor, and posterior parietal systems, extending to occipito‐temporal regions—systems that, in our design, are also implicated in the control‐task target‐detection demands—consistent with a shift toward cue‐driven temporal orienting and sensorimotor preparation in the exogenous paradigm. When these loci are interpreted under canonical resting‐state parcellations, the endogenous‐related effects are biased toward default‐mode and temporo‐parietal association networks, whereas the exogenous‐related differences are biased toward dorsal attention, frontoparietal control and salience/sensorimotor network components, predominantly expressed as negative contrasts relative to the control baseline [23, 69, 70]. This integrated mapping provides a concise network‐level account of how task‐defined endogenous temporal expectation (internally monitored timing supported by a fixed ISI) versus exogenous temporal expectation (externally cued timing) are implemented in the present paradigm, with the exogenous‐related signature captured largely as relative decreases with respect to the target‐detection baseline [25].

Cortical regions were then classified by a result of tensor probabilistic independent component analysis to resting‐state fMRI data [69]. Hippocampal region was fitted with another independent component analysis result [36]. We collapsed multiple activation patterns and found a broad functional separation of the exogenous/endogenous temporal expectation task, which suggests the existence of two distinct activation patterns for exogenous vs. endogenous temporal expectations. Similarly, our sum of activations triggered by the exogenous and endogenous task corresponds to the resting‐state network and conforms to a pattern division of 10 cortical regions showing the full picture of temporal expectations, which emphasizes the interaction between the two modes of temporal expectations at the network level. Mechanistically, the partially dissociable large‐scale profiles observed here suggest that endogenous temporal expectation is supported by internally maintained temporal priors and conditional‐probability updating implemented by default‐mode/temporo‐parietal association hubs together with subcortical timing‐related nodes, whereas exogenous temporal expectation is instructed from online sensory time‐to‐event evidence and is expressed in our contrast primarily as relative reductions in lateral prefrontal–insular–premotor–posterior parietal control systems, consistent with a shift toward cue‐driven temporal orienting and sensorimotor preparation [34, 71]. In turn, this network‐level implementation helps refine current temporal‐expectation frameworks by linking the exogenous–endogenous distinction to probabilistic temporal‐attention computations and mode‐dependent large‐scale network reconfiguration, rather than a single dedicated timing module [32, 72].

Recent work suggests that formal musical training can sharpen contextual representations used for hierarchical prediction during naturalistic music listening [73]. Complementarily, evidence indicates that higher musical training is associated with stronger neural responses to sequence variations, consistent with enhanced prediction‐error signaling during auditory sequence processing [74]. Accordingly, replication in larger, more diverse samples should explicitly quantify musical training and incorporate it as a covariate or stratification factor when modeling individual differences in temporal expectation. The present conclusions should be interpreted in the context of a relatively modest sample size and a demographically homogeneous cohort, which may constrain direct generalization to broader populations. At the same time, the within‐subject design, the consistent behavioral effects across conditions, and the convergent activation patterns across analyses support the internal robustness of the present findings. These findings should be interpreted as preliminary and warrant replication in larger, mixed‐sex, and demographically diverse cohorts [75].

## 5 Conclusion

In the present study, we designed exogenous and endogenous temporal expectations tasks by referring to Nobre and Coull's [10] classification of timing, and explored the related neural substrates. We support that classification of timing of Nobre and Coull's [10], because of the following reasons: 1. when compared the current result to an explicit timing task [8], a reversed pattern of neural systems was found, that the mesial brain regions of the so‐called “default mode network” activated positively and the motor system activated negatively for the exogenous temporal expectations process; 2. When compared exogenous to endogenous temporal expectations task, almost cortical areas could be categorized despite the discrete functions of the brain areas, demonstrating the complementary nature of the two temporal expectation modes.

## Author Contributions

All authors contributed substantially to the study. Chunlin Li, Zhongtian Guan, Zhixi Zhang, and Jingyi Zhao collected the data. Chunlin Li contributed to manuscript drafting. Chunlin Li, Zhongtian Guan, and Jingyi Zhao performed data analysis. Chunlin Li, Jingyi Zhao, Hongbin Han, Dehua Chui, Xu Zhang, Jinglong Wu, Qingyuan He, and Yixuan Yuan designed the MRI methods. Chunlin Li, Xu Zhang, and Wanyi Fu contributed to the conceptualization and psychological framing of temporal expectation. Chunlin Li, Zhongtian Guan, Zhixi Zhang, Jingyi Zhao, and Wanyi Fu revised the manuscript.

## Ethics Statement

This study received approval from the Ethics Committee of the Capital Medical University (2025SY‐045), and all participants provided informed consent prior to their inclusion in the study.

## Conflicts of Interest

The authors declare no conflicts of interest.

## Data Availability

The data that support the findings of this study are available from the corresponding author upon reasonable request.
